# Case Report: Two cases of non-small cell lung cancer with coexistence of *NTRK2* fusion and *EGFR* mutations

**DOI:** 10.3389/fonc.2025.1664782

**Published:** 2025-11-26

**Authors:** Yuping Zhang, Hengming Zhang, Xiufeng Li

**Affiliations:** Precision Pathology Diagnosis Center, Weifang People’s Hospital, Shandong Second Medical University, Weifang, China

**Keywords:** non-small cell lung cancer, molecular testing, *NTRK2*, *EGFR*, fusion and mutation

## Abstract

**Objective:**

To investigate the clinical and pathological characteristics of patients with non-small cell lung cancer exhibiting coexistence of *NTRK2* fusion and *EGFR* mutations.

**Methods:**

Clinical data, as well as histopathological, immunohistochemical, and molecular pathological characteristics, of two patients harboring both *NTRK2* gene fusions and *EGFR* gene mutations were retrospectively analyzed, and relevant literature was also reviewed.

**Results:**

Both patients were women aged 57 and 66 years. The two cases were diagnosed as invasive lung adenocarcinoma, and immunohistochemical staining showed that all tumor cells expressed CK7, Napsin A, TTF-1, and PD-L1. In Case 1, an *EGFR* mutation in the primary lung lesion, coexistence of *NTRK2* fusion and *EGFR* mutation in liver metastases, and concurrent *MET* gene amplification and *FGFR1* gene mutation were observed. In Case 2, the coexistence of *NTRK2* fusion and *EGFR* mutation was detected in the primary lung lesion. The Tumor Mutation Burden (TMB) and microsatellite status were classified as TMB-L and MicroSatellite Stable (MSS), respectively, in both cases. Case 1 received osimertinib combined with savolitinib, had 33 months of follow-up, and achieved a partial response. Case 2 received furmonertinib and achieved a complete response.

**Conclusion:**

*NTRK2* fusion coexisting with *EGFR* mutations is a rare molecular characteristic of non-small cell lung cancer, accompanied by positive PD-L1 expression, and may serve as a promising biomarker for targeted therapy.

## 1 Introduction

Currently, the survival outcomes of patients with non-small cell lung cancer (NSCLC) can be significantly improved through precise molecular typing, attributable to advancements in chemotherapy, targeted therapy, and immunotherapy. Notably, the molecular variation spectrum of patients with NSCLC in China differs from that of the Western population, especially for adenocarcinoma (1, 2). With the development of in-depth discovery technologies and exploration of targeted molecules and tumor-related pathways, an increasing number of genes and loci related to NSCLC treatment have been identified. Multiple variations in targeted therapy-related genes have been discovered, including common variations in genes, such as *EGFR*, *KRAS*, and *ALK*; minority mutations in genes, such as *ROS1*, *MET*, *HER2*, *BRAF*, and *RET*; and rare mutations in genes, such as *NTRK* and *NRG1/2*. In the same case, many variations generally occur exclusively, and the coexistence of these two types of variations has been reported in only a very small number of cases, such as *EGFR* mutations coexisting with variations in *ALK*, *HER2*, *RET*, and *ROS1* genes (3, 4). Recently, the coexistence of these genetic alterations has been increasingly observed—for example, *EGFR* mutations coexisting with *NTRK1* translocations in NSCLC (5). Here, the clinical, histopathological, and molecular pathological characteristics, targeted therapy, and prognosis of two patients with NSCLC exhibiting coexistence of *NTRK2* fusion and *EGFR* mutations are presented to enhance understanding of such rare gene covariations and provide a basis for precise targeted therapy in NSCLC.

## 2 Case description

### 2.1 Case information

Both patients were diagnosed with invasive lung adenocarcinoma at the Precision Case Diagnosis Center of Weifang People’s Hospital. All histopathological diagnoses were reviewed by two senior pathologists. Baseline patient characteristics were collected, including age, sex, clinical manifestations, lymph node and/or distant metastases, imaging findings, pathological examinations (histopathological and molecular tests), treatment, and follow-up information.

### 2.2 Methods

#### 2.2.1 Hematoxylin and eosin and immunohistochemical staining

Specimens from the two cases were fixed in 10% neutral formalin solution, paraffin-embedded, sectioned into 3-μm-thick slices, and processed for hematoxylin and eosin staining and immunohistochemical analysis following the manufacturer’s instructions. An immunohistochemical assay for PD-L1 detection was performed using the Dako Autostainer Link 48 instrument (Agilent Technologies, Singapore Pte. Ltd., Roche Diagnostic Products Co., Ltd, Shanghai, China), and other primary antibodies—including ALK [D5F3, Roche Diagnostics Products (Shanghai) Co., Ltd., Zhongshan Golden Bridge Biotechnology Co. Ltd Co., Ltd, Beijing, China], CK7, Napsin A, TTF-1, P40, CK5/6, CK20, HepPar-1, CK19, CK8/18, Glypican-3, and Ki-67 (ZSGB-BIO Co. Ltd., Beijing, China)—were analyzed using the Roche BENCHMARK XT automated immunohistochemical instrument (Roche Ventana, Roche Diagnostic Products Co., Ltd, Shanghai, China).

#### 2.2.2 Fluorescence *in situ* hybridization assay

*NTRK2* rearrangement was detected using a fluorescence *in situ* hybridization (FISH) assay following the manufacturer’s instructions. The *NTRK2* probe was purchased from Wuhan Kanglu Biotechnology Co., Ltd., HealthCare Biotechnology Co., Ltd, Wuhan, China. Red and green signals were considered separated when the distance between them exceeded the sum of their diameters in tumor cell nuclei, and a separation ratio ≥15% was recognized as positive (separation ratio = number of positive cells/number of total cells × 100%).

#### 2.2.3 Next-generation sequencing

DNA was extracted using a magnetic bead-based FFPE DNA extraction kit (Guangzhou Meiji Biotechnology Co., Ltd., Majorbio Biotechnology Co., Ltd, Shanghai, China). The concentration and quality of DNA were assessed using a NanoDrop 2000 and Qubit 4.0, respectively. DNA sequencing was performed using a targeted high-throughput next-generation sequencing panel for NSCLC [Yuanma Gene Technology (Suzhou) Co., Ltd., MetaGene Technology Co., Ltd, Suzhou, China; National Medical Device Registration No. 20213400525]. TMB and Microsatellite instability (MSI) were detected using the Non-Small Cell Lung Cancer Tissue TMB Detection Kit (Nanjing ShiHe Medical Devices Co., Ltd., Shihejiyin Technology Co., Ltd, Nanjing, China).

## 3 Diagnostic assessment

### 3.1 Clinical features

Both cases involved women.

**Figure 1:**
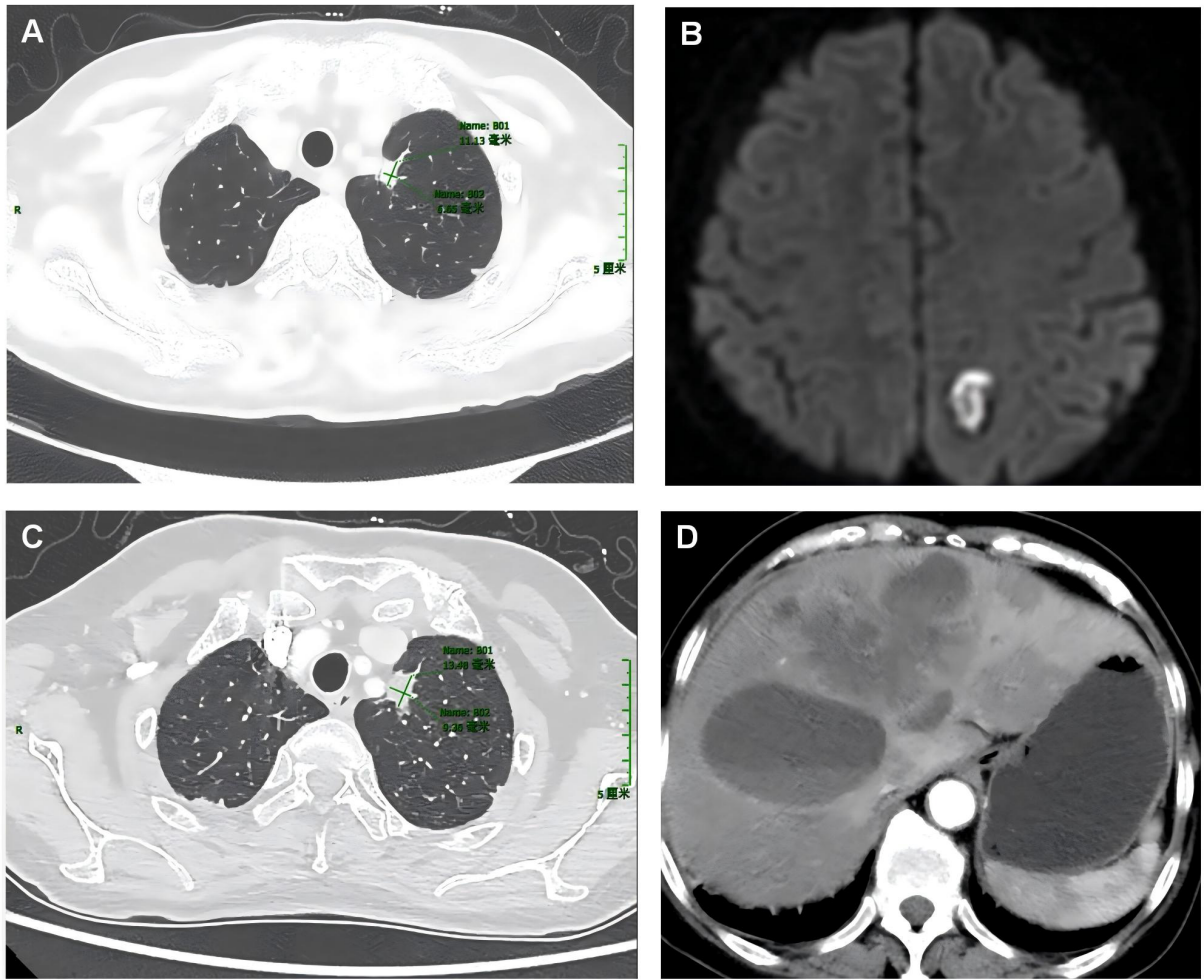
Plain computed tomography (CT) images of the lung and liver lesions, as well as the cranial magnetic resonance images of the first patient. The plain CT scan upon admission showed a mass in the lungs **(A)**, whereas the magnetic resonance imaging of the brain indicated brain metastases **(B)**. In September 2024, plain CT scans of the chest and liver showed lung lesions **(C)** and liver metastases **(D)**.

Patient 1, aged 59 years, was diagnosed at our hospital on October 26, 2022, and presented with an unexplained headache, predominantly generalized cranial distending pain. Plain and contrast-enhanced computed tomography (CT) and cranial magnetic resonance imaging (MRI) showed a solid nodule in the left upper lung lobe with mediastinal lymphadenopathy, multiple ground-glass nodules in both lungs, and multiple intracranial space-occupying lesions. The patient was diagnosed with lung cancer accompanied by brain metastasis (**Figures 1A, B**). No positive signs were found during physical examination. Blood routine test revealed white blood cell count 6.86 × 10^9^/L (normal range: 3.5–9.5 × 10^9^/L), red blood cell count 3.69 × 10^12^/L (normal range: 3.8–5.1 × 10^12^/L), hemoglobin 113 g/L (normal range: 115–150 g/L), and platelets 232 × 10^9^/L (normal range: 125–350 × 10^9^/L). Blood biochemical test revealed total protein 56.5 g/L (normal range: 65–85 g/L), albumin 35.4 g/L (normal range: 40–55 g/L), and urea 2.2 mmol/L (normal range: 2.6–7.5 mmol/L); alanine aminotransferase, aspartate aminotransferase, alkaline phosphatase, glucose, and creatinine were all normal. Tumor markers in blood tests were within normal ranges, including alpha-fetoprotein, carcinoembryonic antigen (CEA), carbohydrate antigen 19-9, and carbohydrate antigen 125. Because of intermittent liver pain, an enhanced CT examination of the chest and abdomen was performed in September 2024 (**Figures 1C, D**), revealing metastases in the liver and left adrenal gland. In November 2024, tumor markers in blood were elevated, including CYFRA21-1 (cytokeratin 19 fragment, 13.86 ng/mL, normal value: 0–3.3 ng/mL), neuron-specific enolase (21.81 ng/mL, normal value: 0–16.3 ng/mL), and CEA (28.81 ng/mL, normal value: 0–5 ng/mL).

**Figure 2:**
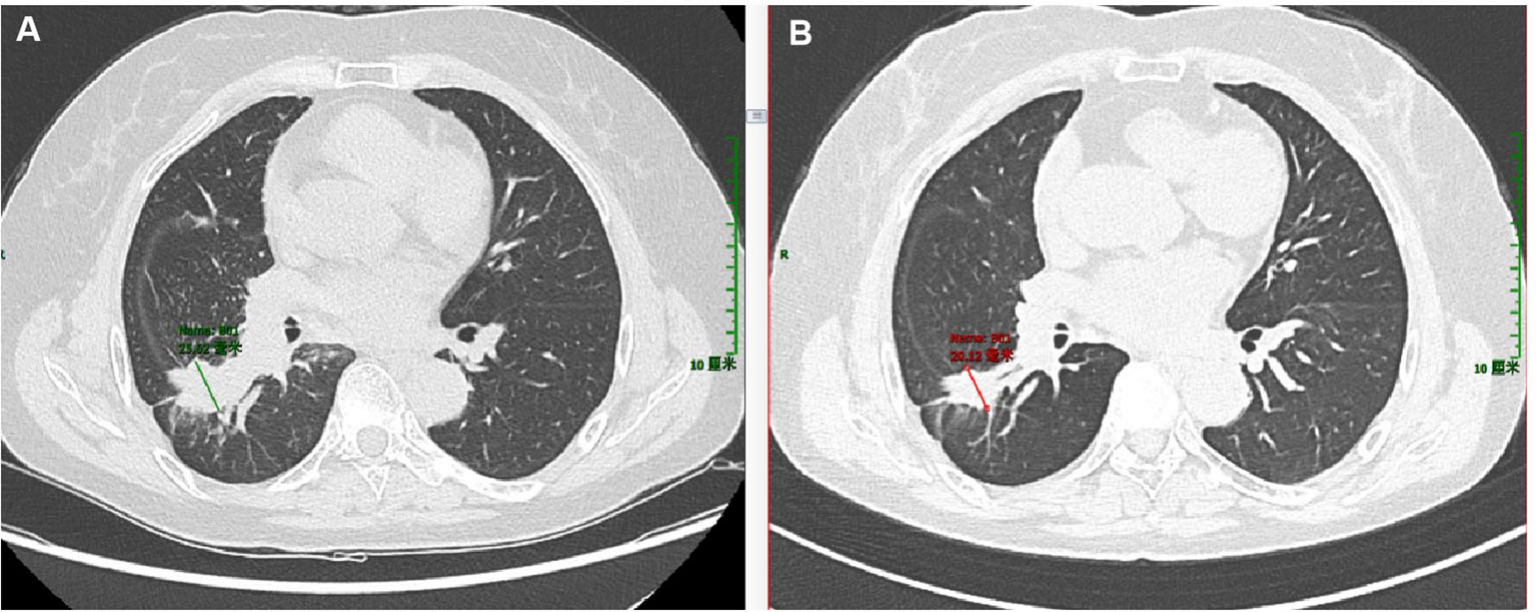
Enhanced computed tomography (CT) images of the lungs of the second patient. **(A)** CT scan of the lungs taken upon admission in April 2024. **(B)** CT scan of the lungs re-performed in November 2024.

Patient 2 was 66 years old. Owing to unexplained coughing and expectoration with a small amount of white, sticky, or yellow phlegm that was difficult to expel, a CT examination was performed at a local hospital. The results demonstrated space-occupying lesions and multiple nodular foci in both lungs. Traditional Chinese medicine treatment was administered, but no improvement was observed. The patient visited our hospital in October 2024. Enhanced chest CT (**Figure 2**) showed a soft-tissue-density mass in the lower lobe of the right lung, 35 mm × 26 mm in size, irregular in shape, with spicules and pleural traction visible at the margin, and moderate enhancement on the contrast scan. No positive signs were found during physical examination. Routine blood tests showed normal white blood cell count, red blood cell count, hemoglobin, platelet count, and normal biochemical results for alanine aminotransferase, aspartate aminotransferase, alkaline phosphatase, glucose, creatinine, urea, total protein, and albumin. Elevated tumor marker levels were observed in blood, including CYFRA21-1 (4.26 ng/mL, normal value: 0–3.3 ng/mL), neuron-specific enolase (25.84 ng/mL, normal value: 0–16.3 ng/mL), and CEA (41.83 ng/mL, normal value: 0–5 ng/mL).

Patient 1 had no history of other health issues. Patient 2 had a 10-year history of chronic bronchitis but did not receive regular treatment. None of the patients had a history of hypertension, diabetes, cardiovascular or cerebrovascular diseases, or infectious diseases, such as hepatitis or tuberculosis, nor had they been in close contact with such cases. Both patients had no history of drug or food allergies; no unhealthy habits, such as smoking, drinking, or drug abuse; no history of trauma, surgery, or blood transfusion; and no family genetic history.

### 3.2 Pathological features

**Figure 3:**
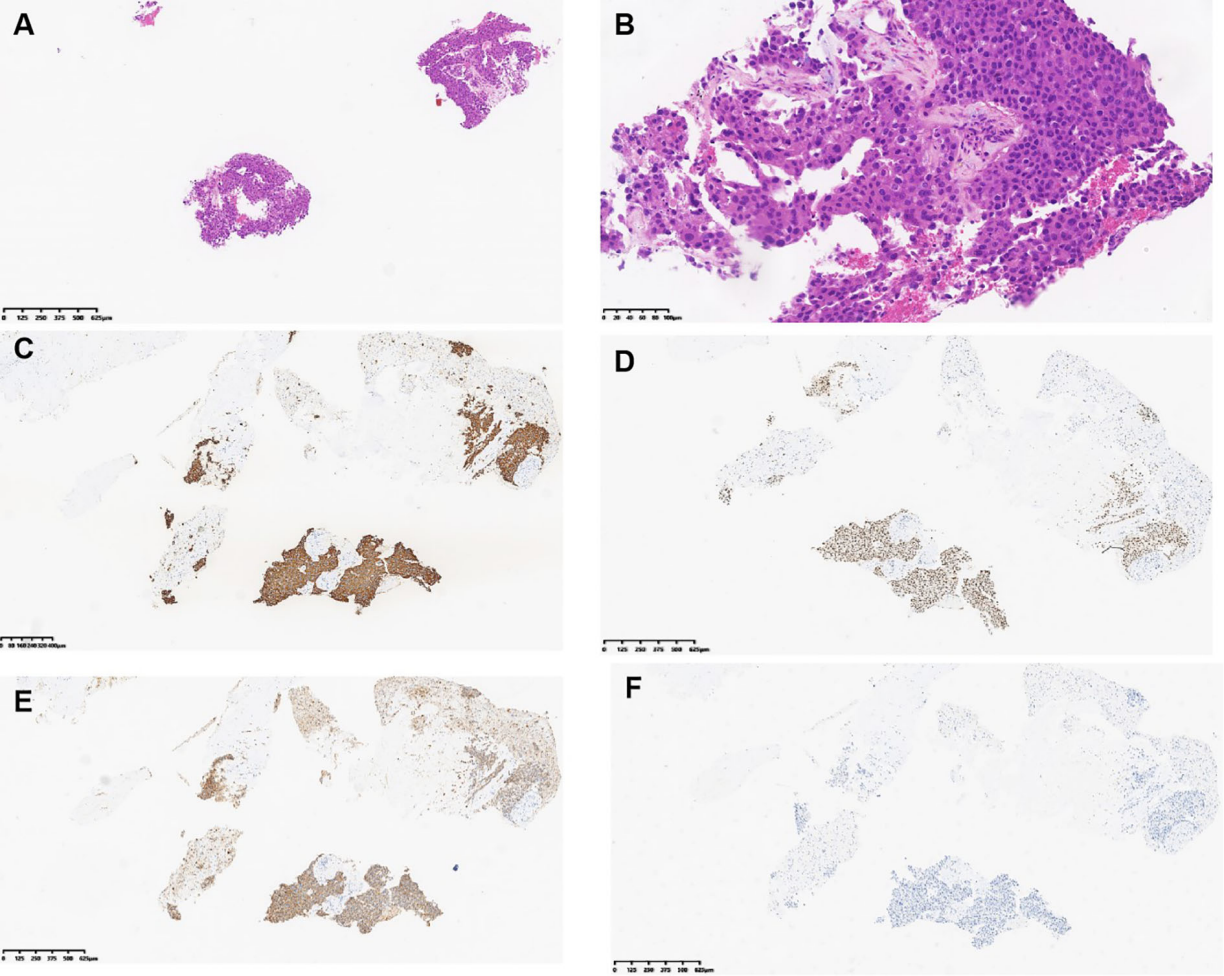
Hematoxylin and eosin (H&E) and immunohistochemical images of the liver lesion biopsy of patient 1. **(A)** H&E staining, magnification ×40. **(B)** H&E staining, magnification ×200. **(C)** Tumor expressed CK7. **(D)** Tumor expressed TTF-1. **(E)** Tumor expressed Napsin **(A)**. **(F)** Tumor did not express HepPar-1. **(C–F)** Magnification ×40.

The morphology of the lung lesion biopsy tissue in Patient 1 in November 2022 is shown in **Figure 3**. The tumor was partially acinar and slightly papillary, with cells of varying sizes, abundant eosinophilic cytoplasm, and rare mitotic figures (**Supplementary Figures S1A, B**). Immunohistochemical staining was positive for CK7, Napsin A, and TTF-1; negative for CK5/6 and P40; and 8% positive for Ki-67. Therefore, the patient was diagnosed with invasive lung adenocarcinoma.

**Figure 4:**
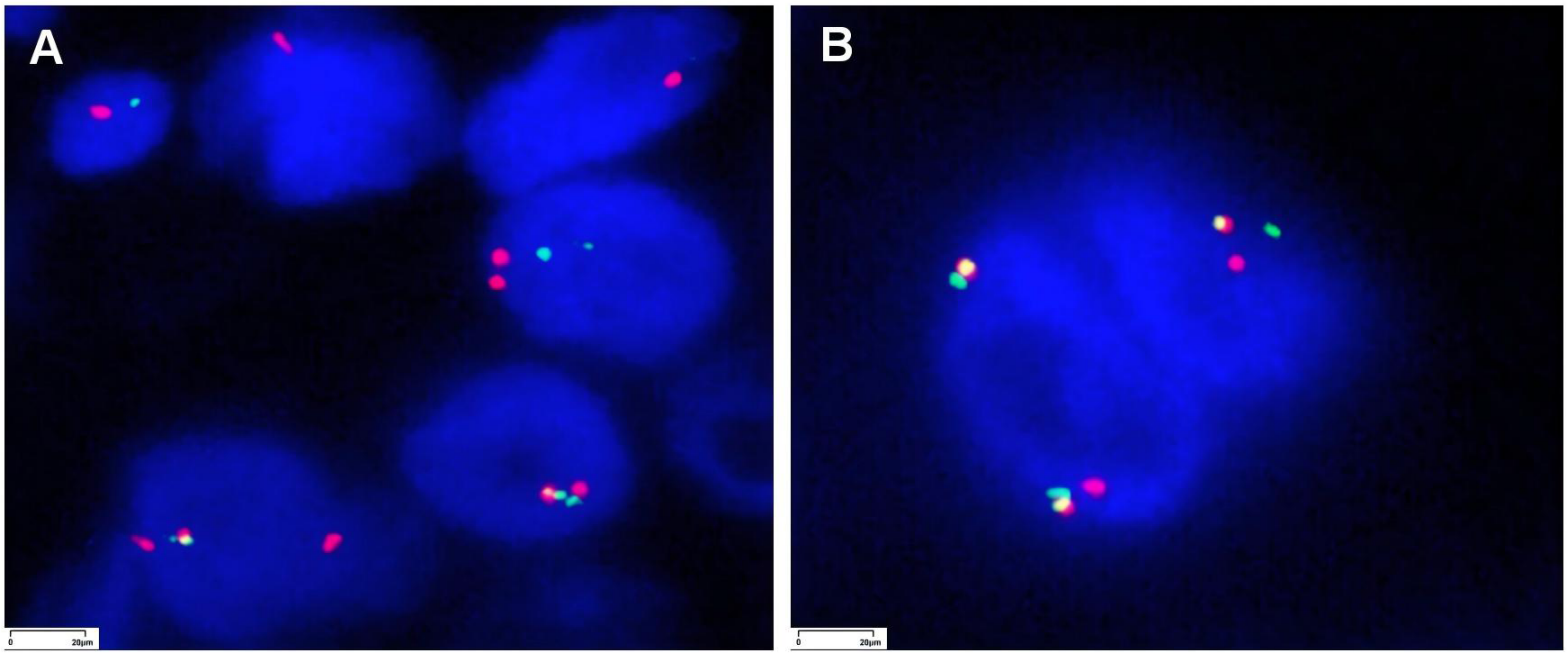
Fluorescence *in situ* hybridization (FISH) images for NTRK testing of the liver tumor in patient 1 and lung tumor in patient 2 (magnification ×1,000).

The morphology of the liver metastases in Patient 1 in December 2024 is shown in **Figure 4**. Atypical cells were observed in proliferating fibrous tissue with marked cellular atypia (**Figures 3A, B**). Immunohistochemical staining was positive for CK7 (**Figure 3C**), TTF-1 (**Figure 3D**), Napsin A (**Figure 3E**), CK19, and CK8/18; weakly positive for CK20; scattered positive for P40; partially weakly positive for Glypican-3; and negative for HepPar-1 (**Figure 3F**). The Ki-67 positivity index was 85%. The PD-L1 (22C3) combined positive score was 50, calculated as follows: (number of PD-L1-positive tumor cells + number of PD-L1-positive tumor-associated immune cells)/total number of viable tumor cells × 100. The ALK (D5F3) companion diagnostic test was negative (tumor cells, positive control + negative control). The patient was diagnosed with lung adenocarcinoma with liver metastasis.

Microscopic observation of the lung lesion puncture tissue in Patient 2 (**Supplementary Figures S2A, B**) showed that the tumor was located beneath the hyperplastic fibers and bronchial mucosa, with tumor cells of varying sizes, abundant cytoplasm, eosinophilia, and rare mitotic figures. Further immunohistochemical staining was positive for CK7 (**Supplementary Figure S2C**) and TTF-1 (**Supplementary Figure S2D**) and negative for CK5/6 (**Supplementary Figure S2E**) and P40 (**Supplementary Figure S2F**). The Ki-67 index was 10%. The PD-L1 (22C3) Tumor Proportion Score (TPS) (%) was 2%, which was calculated as the number of PD-L1 staining-positive tumor cells/total number of viable tumor cells × 100%. The ALK (D5F3) companion diagnosis was negative (tumor cells, positive control + negative control). Ultimately, the patient was diagnosed with invasive lung adenocarcinoma.

In differential diagnosis, pulmonary invasive adenocarcinoma should be differentiated from squamous cell carcinoma and small-cell carcinoma of the lungs. Morphologically, adenocarcinoma typically presents as glandular, sheet-like, and papillary growth; squamous cell carcinoma shows nest-like and sheet-like growth with keratinization and cell bridges visible; small-cell carcinoma presents as sheet-like and nest-like growth with organ-like or compression arrangement, rich blood vessels, small cells, and chromatin in a salt-and-pepper pattern. Regarding immunohistochemical expression, adenocarcinoma expresses CK7, TTF-1, and Napsin A; squamous cell carcinoma expresses P40 and CK5/6; and small-cell carcinoma expresses CK broad (with focal positivity at the nuclear periphery), CD56, Syn, CgA, and CD117. Hepatic metastatic pulmonary adenocarcinoma must be differentiated from primary liver hepatocellular carcinoma and cholangiocarcinoma. These tumors lack specific morphological features and are primarily identified using immunohistochemistry. Metastatic pulmonary adenocarcinoma expresses markers of pulmonary adenocarcinoma, such as TTF-1 and Napsin A. Primary liver hepatocellular carcinoma expresses markers of the liver, such as HepPar-1, whereas cholangiocarcinoma expresses CK7 and CK19; neither of the latter expresses markers of pulmonary adenocarcinoma.

### 3.3 Molecular features

The genetic variations in the two cases are shown in **Table 1**. In FISH, red-green separation and a single green signal were observed in more than 30% of the tumor cells, suggesting *NTRK2* gene rearrangement (**Figures 4A, B**).

**Table 1:** Genetic variations of the two cases.

Patients	TMB value	TMB level	MSI status	Variant genes	Transcript number	Type of gene variation	Nucleotide alteration	Amino acid substitution	Variant region	Variant frequency
Case 1	7.9	TMB-L	MSS	EGFR	NM_005228.5	Missense mutation	c.2573T>G	p.L858R	Exon 21	12.42%
				FGFR1	NM_023110.3	Missense mutation	c.1225C>T	p.H409Y	Exon 9	18.28%
				Gene rearrangement	Variant region					
				NTRK2	IGR (upstream CEP78)~NTRK2: exon 17					
				Gene amplification	Variant region					Copy number
				MET	7q31.2					16.91

				Variant genes	Transcript number	Type of gene variation	Nucleotide alteration	Amino acid substitution	Variant region	Variant frequency
Case 2	6.9	TMB-L	MSS	EGFR	NM_005228.5	Missense mutation	c.2582T>A	p.L861Q	Exon 21	78.35%
				Gene rearrangement	Variant region					
				NTRK2	IGR (upstream DMAC1)~NTRK2 intron 14 IGR (downstream KDM4C)~NTRK2 intron 14					

### 3.4 Treatment and follow-up

#### 3.4.1 Patient 1

On November 10, 2022, and from November 11 to November 30, the patient underwent gamma knife treatment for intracranial metastases. The treatment process proceeded smoothly. A follow-up cranial CT scan showed partial shrinkage of the metastases and edema of the surrounding brain tissue. On November 11, 2022, the patient underwent the first cycle of pemetrexed and cisplatin chemotherapy. On November 16, 2022, the results of the primary lesion gene test indicated an *EGFR* exon 21 mutation, and the patient was administered osimertinib targeted therapy. During the treatment period, the patient did not experience any obvious discomfort and did not undergo regular follow-up. On May 1, 2023, follow-up lung CT showed a slightly smaller lesion area. On July 25, 2023, a follow-up cranial CT scan showed an increased lesion area. Subsequently, the patient underwent gamma knife treatment for the cranial metastases, and the treatment process was smooth. Simultaneously, a follow-up lung CT showed no significant changes in the lesion. Therefore, from October 30, 2023, to January 17, 2024, the patient received four cycles of bevacizumab and sintilimab treatment and continued osimertinib maintenance therapy. The treatment process proceeded smoothly. The patient did not undergo regular follow-up. On April 4, 2024, a follow-up cranial CT scan showed no significant changes in the extent of metastasis. On September 24, 2024, a CT scan showed that the lung lesion had increased relative to previous imaging, and multiple low-density lesions appeared in the liver, which were considered metastases. A left adrenal adenoma was also considered a metastasis. A liver metastasis biopsy was performed on October 23, 2024, and genetic testing was conducted. On November 5, 2024, left adrenal secondary malignant tumor particle implantation treatment was performed, and the process was smooth. Because the genetic test of the liver metastasis revealed *EGFR* exon 21 mutation, *NTRK2* gene fusion, *MET* amplification, and *FGFR1* mutation, on November 25, 2024, the patient was treated with osimertinib combined with certolizumab until January 2025. For economic reasons, bozitinib was used. The patient did not undergo regular follow-up. During the follow-up period until June 2025, a total of 32 months, the patient’s condition progressed slowly.

#### 3.4.2 Patient 2

Based on the genetic test results of the lung biopsy tissue, furmonertinib targeted therapy was initiated on May 9, 2024. The patient did not undergo regular follow-up. On November 11, 2024, CT reexamination showed that the lung lesion had improved. The patient was followed up until June 2025 for 13 months, and the condition was well-controlled.

## 4 Discussion

The NTRK family consists of three members, *NTRK1*, *NTRK2*, and *NTRK3*, which are located at different segments of chromosomes 1q22, 9q21, and 15q25 and encode the tropomyosin-related kinase family proteins TRKA, TRKB, and TRKC, respectively. Fusions of *NTRK1*, *NTRK2*, and *NTRK3* and their partner genes can lead to overexpression of Trk proteins, which in turn activate downstream signaling pathways, such as RAS/MAPK, PI3K/AKT, and PLC-γ, causing transformation, proliferation, and survival of cancer cells. NTRK fusion has been identified as a definite oncogenic driver (6) and is a valuable target for cancer treatment. The frequency of NTRK fusions in common solid tumors is extremely low, with a prevalence of 0.3 to 0.5% (7–9), including in NSCLC.

Recently, the prevalent methods for detecting NTRK fusions have included immunohistochemistry, FISH, reverse transcription polymerase chain reaction (PCR), and second-generation sequencing. However, each method has its own limitations. The European Society for Medical Oncology (6) recommended that FISH, reverse transcription PCR, or second-generation sequencing with RNA-based sequencing panels be used in tumors with frequent NTRK fusions, whereas for tumors with rare NTRK fusions, first-line sequencing (prioritizing RNA sequencing) or initial screening by immunohistochemistry should be performed for NTRK fusion-positive cases. RNA-based second-generation sequencing methods are considered the gold standard for all types of tumors (10, 11).

The most common fusions in solid tumors are ETV6:NTRK3 and TPM3:NTRK1 (12). More than 20 *NTRK1*, *NTRK2*, and *NTRK3* fusion partners have been identified in NSCLC (13), which usually do not coexist with other oncogenic drivers, such as *EGFR*, *ALK*, *ROS1*, *MET*, and *RET* (14). In the present study, *NTRK2* gene fusion was detected in two NSCLC cases, and the unique upstream region of the gene was the fusion location of the partner gene. The partner gene in Case 1 was located upstream of *CEP78*, and in Case 2, the fusion was located downstream of *KDM4C* and upstream of *DMAC1*. Notably, both mutations were accompanied by *EGFR* mutations (p.L858R and p.L861Q). The difference between the two cases was that a secondary *NTRK2* gene fusion was detected in the metastatic lesion in Case 1, whereas in Case 2, it was detected in a primary lung lesion.

A previous study (15) demonstrated that the emergence of *NTRK1* gene fusions may be a mechanism of resistance to epidermal growth factor receptor–tyrosine kinase inhibitors (EGFR-TKIs; such as osimertinib). The Guidelines for Clinical Practice of Molecular Tests in Non-Small Cell Lung Cancer in China (2024) stated that, in practical clinical settings, during the comprehensive analysis of acquired resistance to EGFR-TKIs, detection of NTRK fusion status is of particular importance (10). We did not observe *NTRK2* fusion in the primary lung lesion in Case 1. Instead, we detected *NTRK2* fusions in metastatic lesions after EGFR-TKI treatment (osimertinib). Therefore, we speculate that *NTRK2* fusion may also be a potential resistance mechanism to EGFR-TKIs. However, this conclusion should be verified by further studies with larger clinical sample sizes and basic research.

Currently, the targeted therapeutic drugs approved for NTRK fusion-positive solid tumors (16, 17) include larotrectinib, entrectinib, and repotrectinib. Repotrectinib is also used to treat adult patients with ROS1 fusion-positive locally advanced or metastatic NSCLC and patients with NTRK fusion-positive advanced solid tumors who have received one or two frontline tropomyosin receptor kinase (TRK) or TKI therapies without a satisfactory response or alternative options.

Interestingly, in Case 1, *MET* amplification and *FGFR1* mutations were also observed in the liver metastases. The proportion of primary *MET* gene amplification in NSCLC is 1%–5% (18), which is often secondary to targeted therapy in other driver gene-positive patients with NSCLC and is recognized as one of the important mechanisms related to EGFR-TKI resistance. *MET* amplification occurs in 7%–15% of patients with first-line EGFR-TKI drug resistance and in 5%–50% of patients with second-line EGFR-TKI drug resistance (19). In Case 1, osimertinib, a third-generation EGFR-TKI drug, was used, and *MET* amplification was observed in the secondary metastases, which may be a sign of osimertinib resistance. Therefore, the treatment strategy was changed to osimertinib combined with the MET amplification inhibitor (savolitinib), and the patient achieved a partial response.

*FGFR1* is a member of the FGFR family, which mainly includes four subtypes. In solid tumors, the types of the *FGFR1–4* genes vary. For example, amplification/overexpression is the most common variation in *FGFR1*, and fusion in *FGFR2*; single-nucleotide polymorphisms occur more frequently in both *FGFR2* and *FGFR3*. Previous data showed that FGFR mutations occur more frequently in Chinese than in Western populations, based on data from 10,194 Chinese patients with solid tumors (20), with gene amplification being the dominant form of variation (58.2%). The most common tumor types were urothelial neoplasms (30.5%) and endometrial carcinomas (16.9%). The *FGFR1* mutation (H409Y) in the liver metastases of Case 1 was a somatic mutation, which is a non-hotspot mutation recorded in the database. Although there is currently no clear clinical indication for targeted therapy, abnormal activation of FGFR in lung cancer remains noteworthy, as it may provide a beneficial treatment strategy.

Previous data published by Gatalica et al. (12) showed that PD-L1 expression was positive in 23% of NTRK fusion cancer cases. In the present study, PD-L1 expression was observed in two cases of *NTRK2* fusion. TMB and microsatellite status detected TMB-L and MSS in both cases, which were largely consistent with previous results (12).

The two patients reported in this article were treated with different EGFR tyrosine kinase inhibitors based on the results of genetic testing. After targeted therapy, the patients achieved varying degrees of remission. In Case 1, after disease progression, the gene test results of the metastatic tumors revealed *MET* gene amplification. This indicates that after developing resistance to EGFR-TKIs, the addition of a *MET* amplification inhibitor led to disease remission. In Cases 1 and 2, *EGFR* mutations and NTRK fusions occurred simultaneously. Although the frequency of both occurring simultaneously in patients with lung cancer is very low, targeting both *EGFR* and NTRK fusions together may provide clinical benefits (21), making them a treatment option for patients who have developed resistance to EGFR-TKIs. However, the treatment outcome depends on the specific resistance mechanism. Therefore, more cases need to be collected in the future to further study the efficacy and safety of combined treatment with EGFR-TKIs and NTRK inhibitors.

In conclusion, although NTRK fusions are rare in patients with NSCLC, the detection of NTRK fusions has gained importance with the development of TRK inhibitors. Therefore, we believe that NTRK fusions may represent a viable detection target or a target occurring simultaneously with other pathogenic and/or potentially targetable alterations, providing a promising opportunity for exploring combination therapies in future studies.

## Data Availability

The datasets presented in this study can be found in online repositories. The names of the repository/repositories and accession number(s) can be found below: https://www.ncbi.nlm.nih.gov/, PRJNA1289990.

## Supplementary material

Supplementary Figure 1 Lung tumor hematoxylin and eosin staining of patient 1 (magnification, ×200).

Supplementary Figure 2 The Hematoxylin and eosin (HE) and immunohistochemical images of the lung tumor biopsy of patient 2. **(A)** HE staining, magnification, ×40; **(B)** HE staining, magnification, ×200; **(C)** Tumor expressed CK7; **(D)** Tumor expressed TTF-1; **(E)** Tumor did not expressed CK5/6; **(F)** Tumor did not expressed P40; **(C–F)** magnification, ×100.

## References

[1] Li W Qiu T Ling Y Gao S Ying J . Subjecting appropriate lung adenocarcinoma samples to next-generation sequencing-based molecular testing: challenges and possible solutions. Mol Oncol. (2018) 12:677–89. doi: 10.1002/1878-0261.12190, PMID: 29518290 PMC5928389

[2] Si X Pan R Ma S Li L Liang L Zhang P . Genomic characteristics of driver genes in Chinese patients with non-small cell lung cancer. Thorac Cancer. (2021) 12:357–63. doi: 10.1111/1759-7714.13757, PMID: 33300283 PMC7862783

[3] Chen J Yang H Teo ASM Amer LB Sherbaf FG Tan CQ . Genomic landscape of lung adenocarcinoma in East Asians. Nat Genet. (2020) 52:177–86. doi: 10.1038/s41588-019-0569-6, PMID: 32015526

[4] Gregg JP Li T Yoneda KY . Molecular testing strategies in non-small cell lung cancer: optimizing the diagnostic journey. Transl Lung Cancer Res. (2019) 8:286−301. doi: 10.21037/tlcr.2019.04.14, PMID: 31367542 PMC6626860

[5] Robledano R Lozano MD . An odd dancing couple. Non-small cell lung carcinoma with coexisting EGFR mutation and NTRK-1 translocation: A case report. Diagn Cytopathol. (2024) 52):393–6. doi: 10.1002/dc.25325, PMID: 38634549

[6] Marchiò C Scaltriti M Ladanyi M Iafrate AJ Bibeau F Dietel M . ESMO recommendations on the standard methods to detect NTRK fusions in daily practice and clinical research. Ann Oncol. (2019) 30:1417–27. doi: 10.1093/annonc/mdz204, PMID: 31268127

[7] Romanko AA Mulkidjan RS Tiurin VI Saitova ES Preobrazhenskaya EV Krivosheyeva EA . Cost-efficient detection of NTRK1/2/3 gene fusions: single-center analysis of 8075 tumor samples. Int J Mol Sci. (2023) 24):14203. doi: 10.3390/ijms241814203, PMID: 37762506 PMC10531831

[8] Solomon JP Linkov I Rosado A Mullaney K Rosen EY Frosina D . NTRK fusion detection across multiple assays and 33,997 cases: diagnostic implications and pitfalls. Mod Pathol. (2020) 33:38–46. doi: 10.1038/s41379-019-0324-7, PMID: 31375766 PMC7437403

[9] de Oliveira Cavagna R de Andrade ES Tadin Reis M de Paula FE Noriz Berardinelli G Bonatelli M . Detection of NTRK fusions by RNA-based nCounter is a feasible diagnostic methodology in a real-world scenario for non-small cell lung cancer assessment. Sci Rep. (2023) 13:21168. doi: 10.1038/s41598-023-48613-4, PMID: 38036758 PMC10689426

[10] Pathology Quality Control Center Chinese Society of Pathology Chinese Medical Association Chinese Society of Oncology China Anti-Cancer Association Chinese Society of Lung Cancer Chinese Thoracic Oncology Group . Guidelines on clinical practice of molecular tests in non-small cell lung cancer in China (2024 version). Zhonghua Bing Li Xue Za Zhi. (2024) 53:981–95. doi: 10.3760/cma.j.cn112151-20240527-00338, PMID: 39375078

[11] Dong K Zhu Y Liu X Sun W Yang X Chi K . Feasibility of two-step approach for screening NTRK fusion in two major subtypes of non-small cell lung cancer within a large cohort. Hum Pathol. (2024) 149:39–47. doi: 10.1016/j.humpath.2024.06.003, PMID: 38866255

[12] Gatalica Z Xiu J Swensen J Vranic S . Molecular characterization of cancers with NTRK gene fusions. Mod Pathol. (2019) 32:147–53. doi: 10.1038/s41379-018-0118-3, PMID: 30171197

[13] Overbeck TR Reiffert A Schmitz K Rittmeyer A Körber W Hugo S . NTRK gene fusions in non-small-cell lung cancer: real-world screening data of 1068 unselected patients. Cancers (Basel). (2023) 15:2966. doi: 10.3390/cancers15112966, PMID: 37296928 PMC10252111

[14] Haratake N Seto T . NTRK fusion-positive non-small-cell lung cancer: the diagnosis and targeted therapy. Clin Lung Cancer. (2021) 22:1–5. doi: 10.1016/j.cllc.2020.10.013, PMID: 33272813

[15] Xia H Xue X Ding H Ou Q Wu X Nagasaka M . Evidence of NTRK1 fusion as resistance mechanism to EGFR TKI in EGFR+ NSCLC: results from a large-scale survey of NTRK1 fusions in chinese patients with lung cancer. Clin Lung Cancer. (2020) 21:247–54. doi: 10.1016/j.cllc.2019.09.004, PMID: 31761448

[16] Russo A Lopes AR McCusker MG Garrigues SG Ricciardi GR Arensmeyer KE . New targets in lung cancer (Excluding EGFR, ALK, ROS1). Curr Oncol Rep. (2020) 22):48. doi: 10.1007/s11912-020-00909-8, PMID: 32296961

[17] Farago AF Taylor MS Doebele RC Zhu VW Kummar S Spira AI . Clinicopathologic features of non-small-cell lung cancer harboring an NTRK gene fusion. JCO Precis Oncol. (2018) 2:1-12. doi: 10.1200/PO.18.00037, PMID: 30215037 PMC6132056

[18] Guo R Luo J Chang J Rekhtman N Arcila M Drilon A . MET-dependent solid tumours - molecular diagnosis and targeted therapy. Nat Rev Clin Oncol. (2020) 17:569–87. doi: 10.1038/s41571-020-0377-z, PMID: 32514147 PMC7478851

[19] Leonetti A Sharma S Minari R Perego P Giovannetti E Tiseo M . Resistance mechanisms to osimertinib in EGFR-mutated non-small cell lung cancer. Br J Cancer. (2019) 121:725–37. doi: 10.1038/s41416-019-0573-8, PMID: 31564718 PMC6889286

[20] Wu L Yao H Chen H Wang A Guo K Gou W . Landscape of somatic alterations in large-scale solid tumors from an Asian population. Nat Commun. (2022) 23:4264. doi: 10.1038/s41467-022-31780-9, PMID: 35871175 PMC9308789

[21] Wang JL Wang LS Zhu JQ Ren J Wang D Luo M . Survival benefit of combinatorial osimertinib rechallenge and entrectinib in an EGFR-mutant NSCLC patient with acquired LMNA-NTRK1 fusion following osimertinib resistance. Respirol Case Rep. (2022) 10:e01054. doi: 10.1002/rcr2.1054, PMID: 36258694 PMC9574602

