# Supplementary figures and images for: Case Report: Two cases of non-small cell lung cancer with coexistence of NTRK2 fusion and EGFR mutations
Source: Front Oncol. 2025 Nov 26;15:1664782. doi: 10.3389/fonc.2025.1664782 (PMC12689392; doi:10.3389/fonc.2025.1664782)

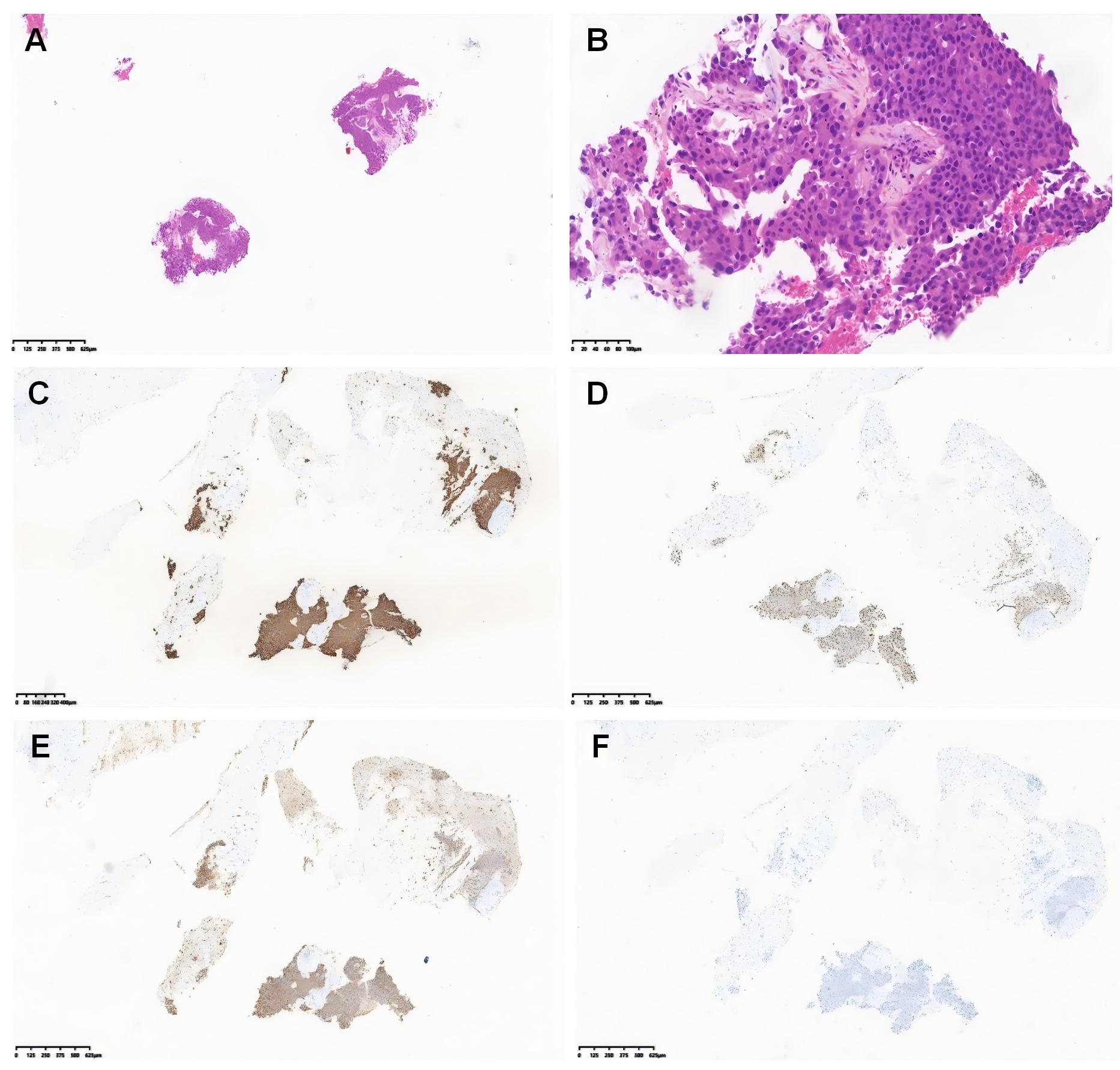

Supplement: Supplementary file 1 [file Image3.jpeg]

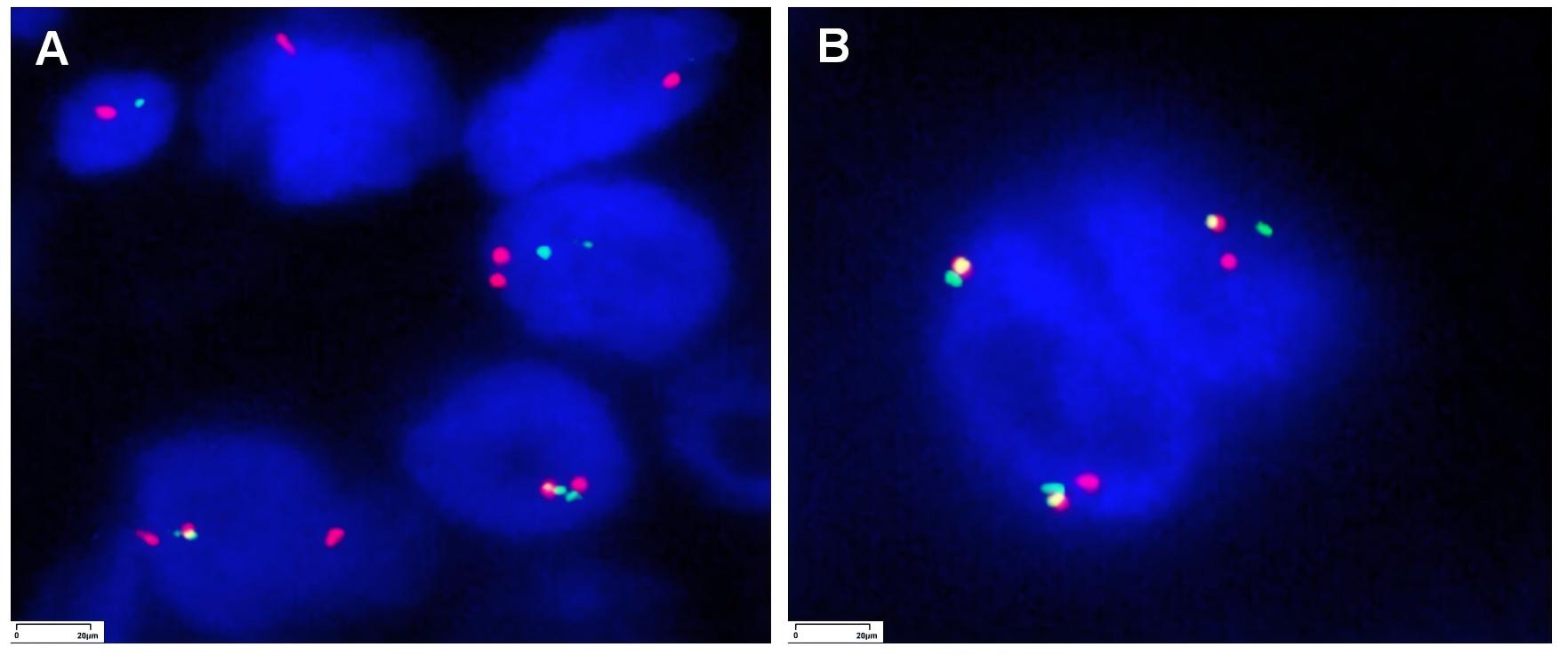

Supplement: Supplementary file 2 [file Image4.jpeg]

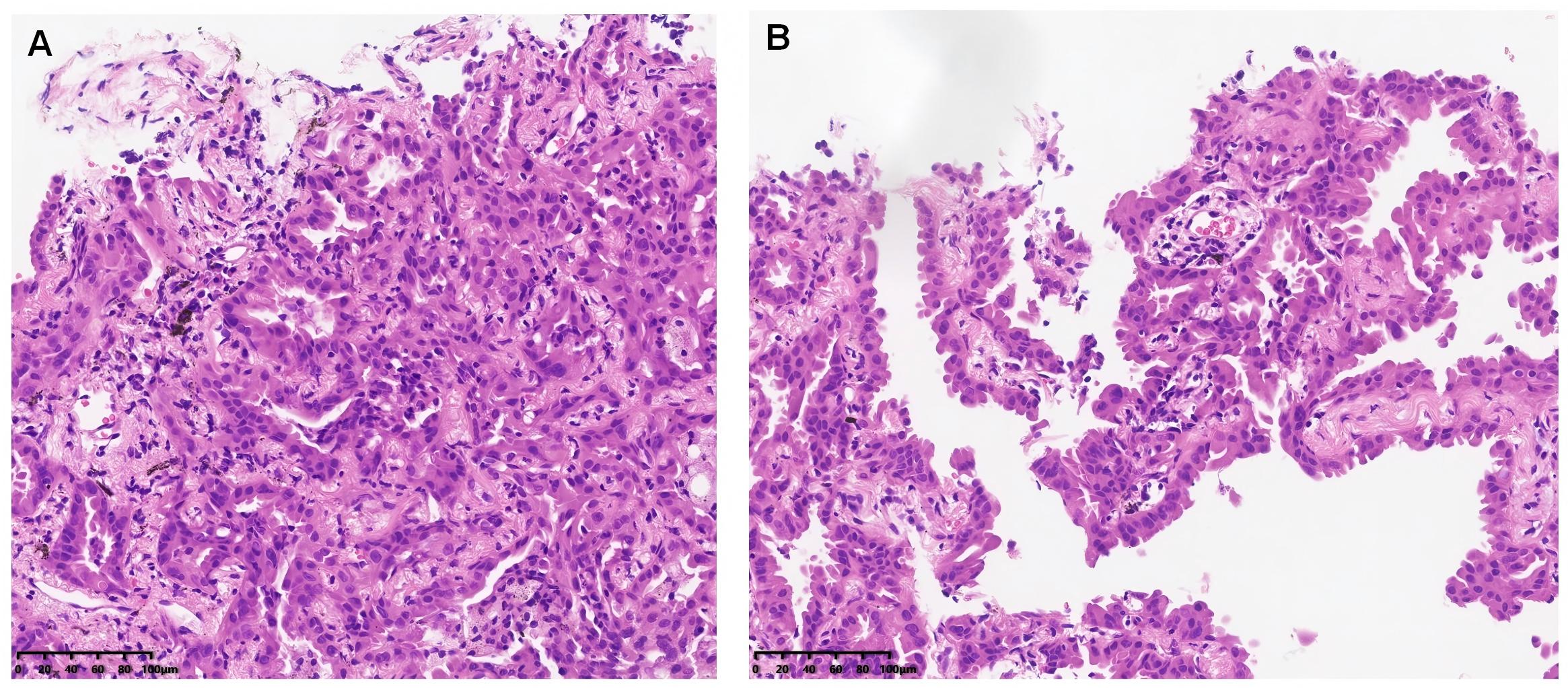

Supplement: Supplementary file 3 [file Image5.jpeg]

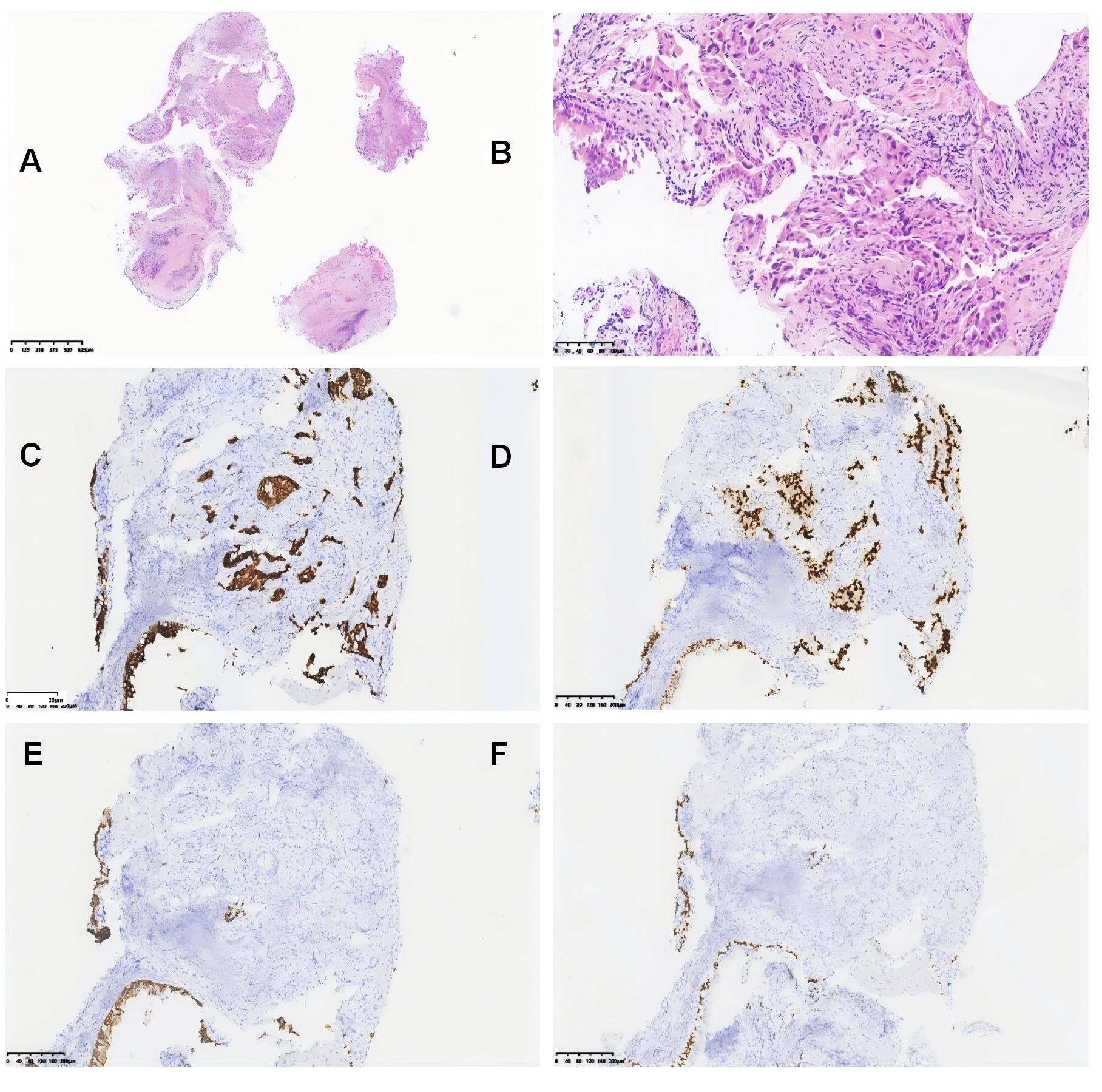

Supplement: Supplementary file 4 [file Image6.jpeg]

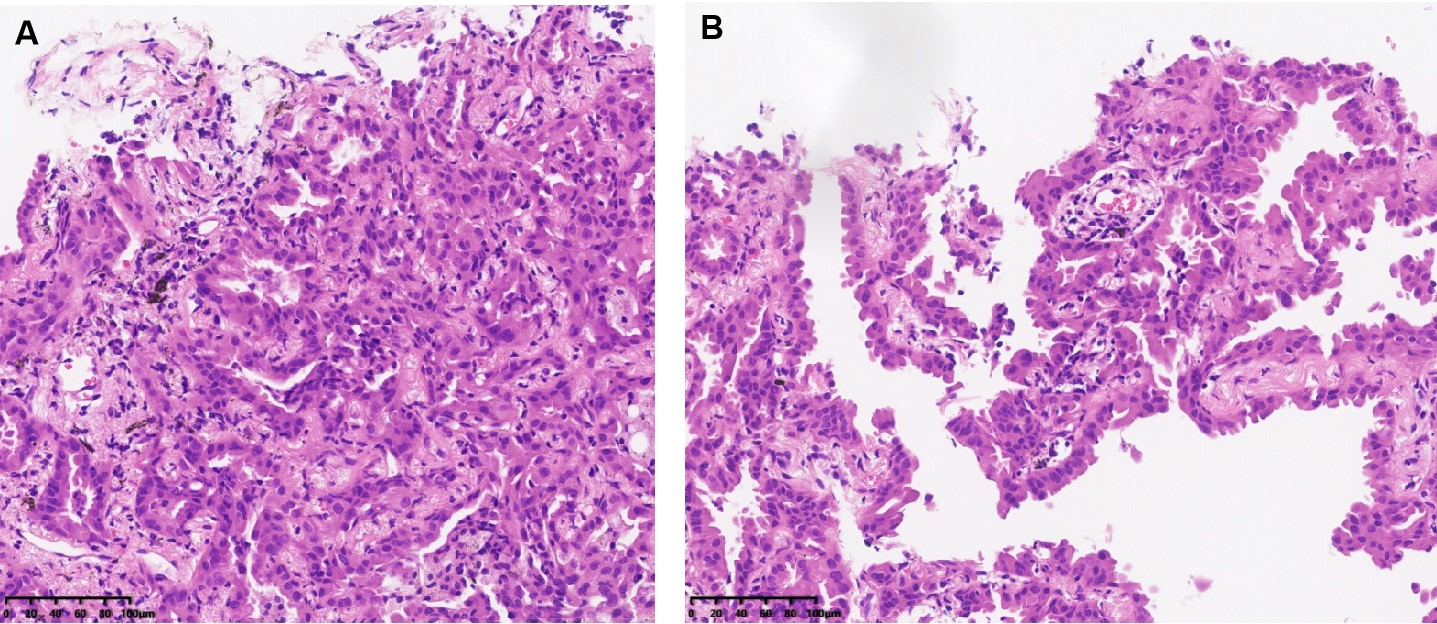

Supplement: Supplementary Figure 1 — Lung tumor hematoxylin and eosin staining of patient 1 (magnification, ×200). [file Image1.jpeg]

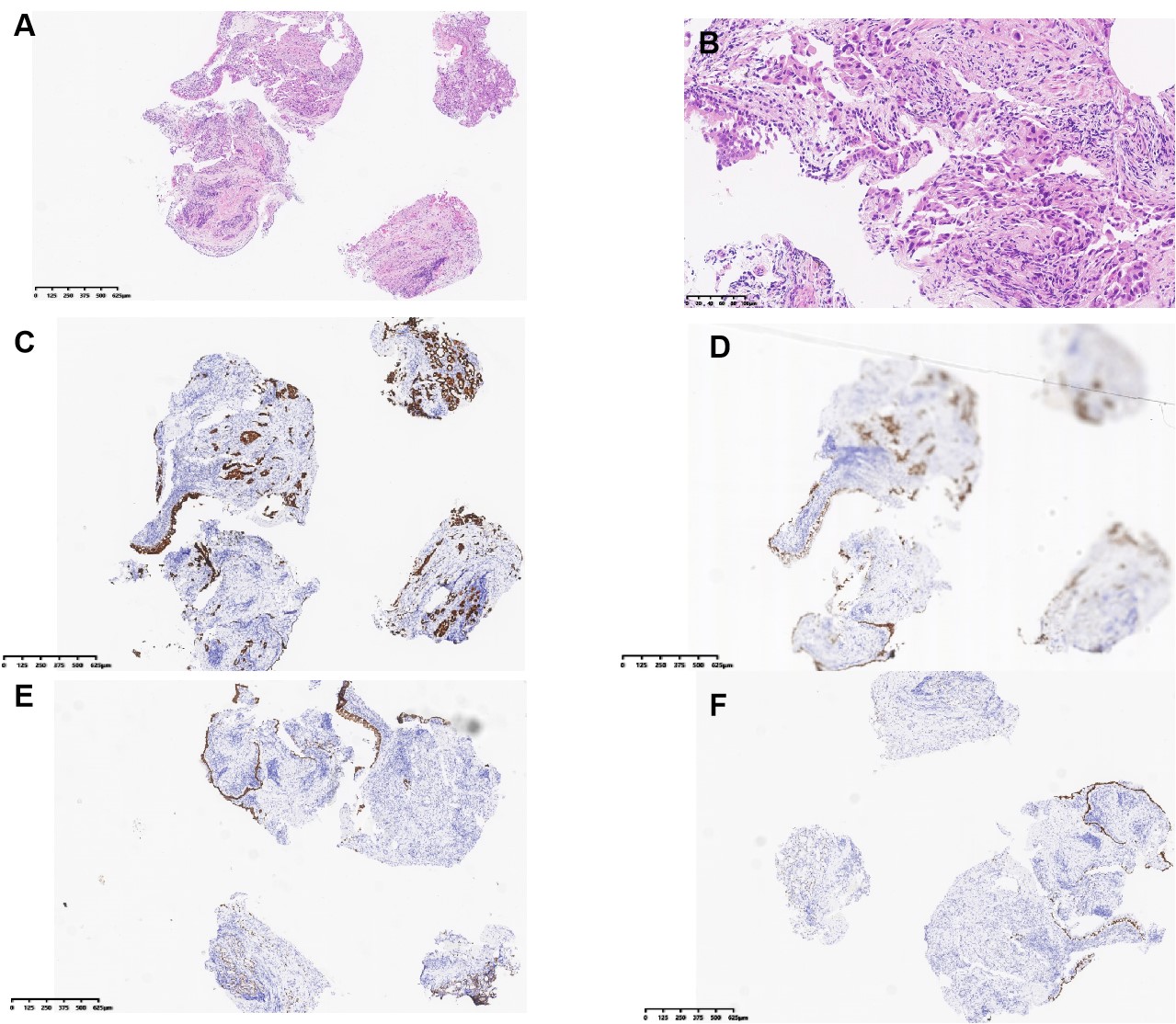

Supplement: Supplementary Figure 2 — The Hematoxylin and eosin (HE) and immunohistochemical images of the lung tumor biopsy of patient 2. (A) HE staining, magnification, ×40; (B) HE staining, magnification, ×200; (C) Tumor expressed CK7; (D) Tumor expressed TTF-1; (E) Tumor did not expressed CK5/6; (F) Tumor did not expressed P40; (C–F) magnification, ×100. [file Image2.jpeg]
